# The Swedish Municipal Food Distribution Service to the Elderly Living at Home as Experienced by the Recipient’s Relatives

**DOI:** 10.5539/gjhs.v5n6p12

**Published:** 2013-07-23

**Authors:** Zada Pajalic

**Affiliations:** 1 School of Health and Society, the PROCARE Group, The Network for Eating and Nutrition, Kristianstad University, Elmetorpsvägen, Kristianstad, Sweden

**Keywords:** elderly, food distribution, homebound, grounded theory, relatives

## Abstract

The municipal Food distribution service (FD) to the elderly living at home is a part of the public social and care service in Sweden, The objective of this service is to ensure proper food intake for persons who are unable to do their own shopping, and prepare their own meals. The foremost reasons for the need of the FD service are in situations where there are illness related physical or psychological limitations. This means that the Swedish welfare system takes on the responsibility for its citizens when they have a legal social related need of care. Further, according to the Swedish social legislation, children or other relatives have no legal obligations to take care of their parents or elderly disabled relatives. This also means that the children or relatives of elderly people requiring social support have no legal right to be involved in the evaluation procedure of need assessment or the outcome of any social and care services granted by the Swedish social welfare system.

The aim of the present study was to gain insight into how the relatives of elderly people living at home in Sweden experience the municipal service of ready-made meals distributed daily.

The data was collected using in-depth interviews with relatives of elderly persons who use the municipal food distribution (FD) service (n=8). The transcribed interview material was analysed using the grounded theory method.

The findings of this study revealed that the relatives of the municipal FD service recipients advocate for a food preparation service in the home of the recipient rather than the distribution of ready-made meals from a central kitchen. The results also revealed that the participating relatives felt frustrated by the legal limitations that make it impossible for them to influence the municipal FD service. The findings in this study also indicate that relatives should be considered as a resource in this matter and could actively participate, and have a positive influence on the quality of the municipal FD service for the elderly. The results of the study could have implications for nursing, public administration and gerontology.

## 1. Background

The municipal food distribution (FD) service is a part of the public social and care service in Sweden (Pajalic, Westergren, Skovdahl, & Persson, 2013). The public social and care service in Sweden has a statutory obligation which is regulated by two acts of parliament: The Health and Medical Service Act and The Social Service Act (Grönwall & Holgersson, 2000; Raadu, 2011). The municipalities in Sweden (n=290) have the responsibility for the FD service. The fundamental requirement for a person to be granted the municipal FD service is that they are unable to do their own shopping and prepare their own meals, and that the foremost reasons for this situation are illness related physical or psychical limitations (Porter, 2007). Before a person can be granted the FD service from any municipality, an assessment of their individual requirements has to be made by a public home care officer (Pajalic, Persson, Skovdahl, & Westergren, 2012). The organisation of the public social and care service in Sweden often differs from other welfare states, primarily due to the fact that the public social and care service is based on the State instituted legal obligation to help persons in need and the obligations are financed by taxation (Elmér, 2000). In Sweden, the relatives, including their children, of a person requiring any type of social assistance have no specific legal obligations or care responsibilities towards their next of kin. These matters are the responsibility of the State through the municipalities (Szebehely, 2003). The charges for public social and care services are regulated and based on the receiver’s ability to pay. However all persons covered by the act are obliged to pay a maximum of 10% of the total costs (Elmér, 2000). The Swedish social politics and welfare system aims to secure citizens’ good welfare and social security. Social security means that each citizen is legally entitled to have their personal needs met through a social agency when they find themselves unable to fulfill their needs themselves. Any citizen may receive state assistance to attain a reasonable standard of living. The concept of a reasonable standard of living is determined by making a comparison with what a normal citizen can generally afford (Hetherington, Anderson, Norton, & Newson, 2006). This concept is not conceptualised in the text of the law but there is a paragraph in a government bill stating that the definition of a “reasonable standard of living” means that citizens may expect to have their needs of assistance met by a State social agency (Lagergren, 2002). The basis for defining what is a reasonable standard of living is to estimate what a low income town dweller can afford in comparison with what the applying citizen has available to cover their cost of living. Social support and assistance for citizens needing help is a task for the municipality. In accordance with the Swedish Social Service Act (Elmér, 2000), society has a legal responsibility for the social care of its citizens who are in need of help, which means, that the relatives and children of the person in need of help relinquish, in law, any legal responsibility for the welfare of the person who is receiving assistance under the Swedish Social Service Act (Gustafsson & Andersson, 2004).

A literature review revealed that there is no existing research material focusing on how the relatives of elderly people living at home experience the municipal FD service. This knowledge is important in order to provide more information about the issue, and in turn it could help public home care officers in their needs assessment work.

The aim of the present study was to gain insight into how the relatives of elderly people living at home in Sweden experience the municipal FD service.

## 2. Method

### 2.1 Context

The study was conducted within an average-sized municipality in southern Sweden, totalling about 80 000 inhabitants. Approximately 650 elderly people, living in the municipality, used the municipal FD service. Although this number could vary, on a daily basis, since some recipients only used the municipal FD service on certain days of the week. The meals were produced in a local municipal kitchen. The menu consisted of two hot dishes each day and an extraordinary meal once a month. The most common meal delivered was lunch, which corresponded to 30% of the clients’ daily nutritional requirement. The possibility to also have evening meals delivered also existed, but was not commonly used. The lunch also included raw vegetables and fruit or dessert, all inclusive. Assistant nurses or taxi drivers were responsible for the delivery of the meals. The meal box was supposed to be delivered personally to the recipient, that is to say “into their hands”, and not simply left outside their door. The municipal FD service is part of the Swedish welfare system and that means that people who have the need for ready prepared meals can retain this service following a needs assessment. According to the Swedish welfare system and The Social Service Act, Swedish society has the responsibility to take care of its citizens, in among other ways, by offering a municipal FD service when they are unable to do their own shopping and prepare their own meals and in that way ensure them a reasonable standard of living. The foremost reasons for requiring help with prepared meals are illness related physical or psychical limitations. By the implementation of the social service legislation the relatives and children of the recipients are relieved of any legal obligation to take care of the elderly or disabled care recipient. Also they have no legal right to be involved in any needs assessment evaluations or decisions made by the home care officers on the municipal care recipient’s behalf.

### 2.2 Participants and Data Collection

The participants in this study were selected through purposeful sampling (Patton, 2002); the selection criteria were that the participants should have a parent or relative receiving the municipal FD service. The data was collected using in-depth interviews with relatives of elderly persons who used the municipal FD service. (n=8). Saturation was reached after 5 interviews and analyses, after which three additional interviews were conducted to ensure the quality of the information. Five of the informants were children to elderly persons who used the communal FD service, one was good friend of one of the recipients of the service and two were sons. A pilot interview was conducted to test the validity of the questions. All the interviews took place at the informants’ work place. The questions (Table 1) were given to the participants at the start of the interview which lasted between 30-90 minutes. The data was collected using a tape-recorder. All the interviews were transcribed verbatim and the constant comparison analysis began simultaneously.

**Table 1:** Examples of questions related to the municipal FD service used in the individual interviews

Can you describe the municipal FD service as you experience it?
What are the strengths or weaknesses of the FD service from your point of view?
What is most important point to satisfy with the FD service from your perspective?
How would you wish to see FD the service organised?

### 2.3 Data Analysis

The data was analysed using grounded theory (Charmaz, 2006; Glaser, 1978). The researcher listened first to the tapes, read through the transcripts to get a feeling for the whole, extracted the facts and then applied a substantive coding technique. Substantive coding was performed in the following steps. Firstly the transcripts were coded line-by-line (in vivo) using the phrases used by the participants’. Then the phrases were shortened and developed into a code that captured the main idea of what the participant had said. Coded phrases were reduced by grouping them together into similar codes and phrases. Then the terms were evaluated into concepts. The next step was the naming of the categories followed by theoretical coding in order to isolate the concepts and connect them. This was followed by seeking new connections of categories and the definition of the subcategories. The emerging of defined categories was linked. The next step was the definition of the core category and the identification of the basic social process by the leading core category. Finally, an explanatory framework was created which led to the derivation and development of a substantive theory (Pajalic, 2013a).

### 2.4 Ethical Considerations

The study was performed in accordance with the Helsinki Declaration (Saif, 2000), and has been examined by the Regional Ethical Review Board in Lund, Sweden (LU09/365). All participants gave their informed consent to participate in the study after having been presented with detailed information about the study and their own participation. They were also informed that they had the right to terminate their participation at any time without it having any consequences for them.

## 3. Findings

### 3.1 The Derivation and Development of a Substantive Theory

The participants agreed that they would prefer the food preparation to be done in the recipient’s home rather than centrally prepared and distributed. They motivated their preference with the explanation that food making at home creates a real feeling of homemade food and a real feeling of being at home. The participants considered that the municipal FD service distributed meal portions that are either too large or too small, in particular, energy and protein enriched food. One of participants noted that they felt that it was not acceptable to have so large differences in portion sizes from day to day. One of participants explained: *“I complained that the portions were too small or too large but nobody listened to my opinion”*. The participants were in agreement that they have positive experiences from their contact with the municipal kitchen staff when they have had the need to ask questions related to the delivered meals.

For the municipal FD service, summer brings difficulties due to it being the major vacation period requiring substitute personnel and during this period it is common that the wrong meals are delivered which causes problems as the contents of the meals are often specific to the individual dietary requirement of the recipient. Further the participants described some more practical problems such as the problem for the meal recipients to get to their front door to open it for the FD service in such cases were the recipient is handicapped in some way. The participants expressed that they would like to take part in developing the meal service from their own perspective and how they should prefer to see the service administrated. As the participants expressed it: “*The mealtime should embrace as much a sense of homeliness and family feeling as possible at the moment it is just simply feeding”*. Criticism was leveled at the timing of the delivery of the meal boxes which can vary by about one hour and that the boxes were difficult to open, and further, that the food was not always easy to chew and that it is not tasty. The participants expressed concern that no one within the municipal food distribution system has the responsibility to provide weight control of the food recipients for use as a parameter to indicate actual and consequent food intake. The participants were agreed that the administration around the distribution of the meals could strengthen the receiver’s isolation and sense of loneliness at mealtimes, especially those who were immobile. One of the participants expressed it as: *“It would be interesting to regularly take the Body Mass Index all those recipients, I believe that the results would be frightening”*.

### 3.2 Taste of the Food in the Meal Boxes

The majority of participants noted the following: Their relatives described the food in meal boxes as being tasty and that the courses often vary, and that the food was tastier when eaten in the middle of day. In some cases the recipients contacted the personnel at the kitchen and expressed their gratitude for the quality of the food which they described as being just like home-made food and that the soups were good. On the other hand the participants expressed their dissatisfaction with the fact that their relatives were sometimes not satisfied with the food delivered on the grounds that it was badly flavoured, often cold, watery, that there was too much tomato paste, the potatoes did not taste nice, there was too much salt, the vegetables were watery and sometimes there were strange sauces not appreciated by older persons. One of participants expressed his frustration over his experience of the quality of the delivered food by asking: *“Do the meals contain sufficient vitamins?”*. It appears from above that the participants expressed more critical views about the municipal FD than did their recipient relatives. They expressed that the food was not tasty and that is the reason why their relatives often throw away uneaten food from the meal boxes. One of the participants said:” *I throw away approximately a third part of the meal portion my mother receives, it depends on how the food tastes”*.

The participants described that food supplied in the meal boxes does not have the same quality during the summer and vacation periods when there are substitute personnel working in the central kitchen. They question the nutritive value of the delivered food and some participants believed that is more about supplying sufficient quantity to avoid the recipients being hungry. One of participants described it as: *“I taste the food sometimes and it is clear that eating it is not going to be a fantastic experience”*. Most of the criticism they directed was towards the fact that the vegetables were often over cooked which resulted in the loss of all the nutrition.

### 3.3 Variation of Food

The participants described the variation of the food as being quiet satisfactory. One of the participants expressed it as: “*I always used to look for the nutritional content and I believe that there is a lot of fish which I believe to be good*.” and another explained it as: *“I think that the food is good”*. The participants suggested that it would be best if the recipients prepared a list of the dishes they would like. As two of them said: *“Let us have a preference list of dishes not that are on the ordinary menu, and how do I tell the FD what I don’t want”*.

### 3.4 Time for Delivery of Meal Boxes

The timing of the delivery of meal boxes was discussed and both the recipients and their relatives had the same experiences such as: that the meal boxes were not delivered punctually, that the delivery came either too early or too late, the delivery of food by assistant nurses was punctual, the promised time of delivery is not so carefully followed, that the time gap between breakfast and lunch should not be too short to allow the recipient to digest one meal before another is served. Some of the recipient’s wished that the Sunday lunch delivery could be done later so that they could go to church first. One of participants expressed it as: “*I was upset that the food arrived early making the time between breakfast and lunch too short which didn’t allow my father to digest his breakfast before eating lunch”*. Another participant expressed it as: *“Receiving the lunch time meal box at 11:30 after having had breakfast at 10:00 brings the two mealtimes too close together*.

### 3.5 Social Aspects of the Municipal FD Service

All of the participants highlighted the social aspects of the mealtimes and that the methods of the municipal FD service strengthened the sense of loneliness and isolation among elderly recipients living alone and without frequent contact with other people. One of participants said:*” People are exposed when living at home alone”*. They described the difficulties for relatives to attend their nearest day centre or restaurant. Many of the participants tried to compensate the social aspects of mealtimes by visiting the recipient but often they lived far away and all of them had jobs which meant that they did not often have the opportunity to give as much social support to their relatives as they would have wished. One of them expressed it as: *“It would be nice if mother had the possibility to eat her meals together with other people”* Another participant expressed it as: *“My mother eats alone”*.

### 3.6 There is No Responsible Person Who Can Give Answers to Questions from Relatives of the Recipients, Related to The Municipal FD Service

The participants described that they have the possibility to contact the public home care officer if they have some questions about the municipal FD service and the meal boxes. None of the participating relatives could remember that they had ever met or a public home care officer or heard that they made home visits to municipal FD recipients. All of them agreed that it is important that the responsible public home care officer is available to answer questions from relatives related to the municipal FD service. One of the participants expressed this problem as: *“It is not easy to know, as an elderly person, whom to talk to and have the possibility to ask questions to the responsible FD service personnel in the municipality. This for me is to marginalise the recipient”*. One of the participants believed that the elderly population, in general, are not used to raising their problems with an authority as they have a fear of authority and believe that their situation could become worse if they raise problems. Often they do not expect to be met positively.

## 4. Discussion

### 4.1 Methodological Considerations

The findings of this study can be evaluated in terms of trustworthiness, credibility and dependability (Glaser, 1978; Lincoln & Guba, 1985). Trustworthiness was used to ensure conscientiousness and honesty from the beginning of the research process and throughout the different steps of the analysis, in order to finally present a clear and complete picture through identifying statements from the participants, while, at the same time, making a continuous comparison between the data, codes and categories throughout the analysis (Charmaz, 2006; Fridlund, 1998). Credibility was assured by presenting views from different participants who were purposely chosen in order to capture different experiences. The purpose of research using a grounded theory design is to achieve an understanding of the social processes that clarify an individual’s actions in relation to others (Glaser, 1978). Further, credibility was ensured by the fact that the sample of participants was representative and that the method of data collection was relevant to the aim of the study. As the participants themselves applied to participate in the study, the author was aware of the possibility that this might have had an influence on the theory generated, so there was reason to question if those participants who chose to participate in this study might think differently in these matters than those relatives who did not participate. Dependability was assured by the fact that the same researcher, the author ZP, carried out all the interviews, analyses and transcriptions. The use of a tape-recorder and verbatim transcripts, as well as referring back to, and re-reading the transcripts during the analysis process allowed the researcher to remain close to the text. The citations make it possible to assure conformability. The author’s pre-understanding was perceived as strength, although there might be a risk of giving augmentation to preconceptions. In order to minimise this risk the researcher attempted to be as open-minded as possible during the interviews. Control was ensured only after the implementation of a pilot interview, and was reached through continuous movement back and forth between the whole and the parts during the analysis process as well as confirmation in the data. Quality criteria for generated theory seems to be fulfilled: it fits with the data, it is relevant and adaptable (Pajalic, 2013a).

### 4.2 Discussion of the Results

The present study showed that the participants preferred a service in the form of food preparation in their relative’s home. They believed that this form of service at home would give multiple positive effects such as the possibility for the recipient to be involved in the preparation of the food, and that the aroma from the preparation would stimulate the recipient’s appetite, and further that the presence of others would help fulfill other social aspects. Another study (Pajalic et al., 2012) has shown that this form of service was preferred even by the decision makers responsible for municipal FD service. A further study (Attree, 2001) has shown that the social aspects related to mealtimes can influence a recipient’s experience of the food service being good or poor. Further, it has been pointed out in other studies that a municipality’s economy is an important factor that influences the organisation of their social and care services (Bergmark, 2000; Pajalic, 2013a).

The present study shows that the participants had difficulties in expressing their observations and viewpoints, related to the municipal FD service, to the involved officials. Further it was shown that the participants were engaged in their desire that their relatives should receive a high quality FD service. Another study has also shown that various perspectives and collaboration between the various professionals involved in the municipal FD service is important in order to improve and adjust the service to meet elderly peoples’ nutritional needs (Pajalic et al., 2012a; Zeitz et al., 2011). However, the present study revealed that there were no personnel within the municipal FD service with an overall insight and responsibility for the whole FD chain and discrepancies in the legislation make it difficult for collaboration between all those involved in the FD service and further, that there is a need to develop knowledge in nutrition. This study revealed the extraordinary fact that there appears to be no one among the FD service personnel, in the municipality used for the study, who knows what happened with the meals after they were delivered to the recipients nor, how much of the food is actually eaten and if it is correctly stored before use or the portions remaining after the meal are correctly stored (Pajalic, 2013b).

This study has shown that the participants questioned the quality of the distributed meals and how this impacted on the recipient’s nourishment intake. This question was supported in another study where it was shown that there are no basic medical control procedures, such as weight or Body Mass Index control, made during the needs assessment process for the municipal FD service. Further, the same study has also shown that only when undernourishment is identified do the FD service administrators involve the services of registered nurses. So the prevention for undernourishment is not routine, within the municipal care system, for elderly persons living at home (Pajalic et al., 2012b). This means that there is no input information regarding nourishment or structured routines to follow up the effects of this service on an elderly person’s health and nutrition status. This can lead to a situation where it will be difficult to follow up the result of the recipient’s food intake if their status at the beginning of their receiving the FD service is unknown the result of which is that it not being possible to register if the outcome of using the FD service is positive or negative for the recipients health Further, it appears that the involved professionals only take responsibility for their particular part in the FD chain and that no one person appears to have an overall picture of the service and its results. Those who appear to take more responsibility, in relation to their competence and responsibility level, are the kitchen personnel and the distributors of the meals. This gives the impression that elderly people living at home do not have access to social and care support at the same high level as those who live in institutions for the elderly. Further, there is a risk that the differences within the governing legislations lead to a focus on structure rather than autonomy and the objective to assure a well-adjusted individual service. The recipient’s individual needs should always be in focus and this requires collaboration, to develop the municipal FD service, among all those involved, which, includes the recipient’s relatives and the FD consumers (Pajalic, 2013b; Samuelsson & Wister, 2000). The results in this study were strengthened by another study where the importance of nutritional assessment for elderly persons living at home was shown (Iizaka, Tadaka, & Sanada, 2008). The observations and experiences of the recipients families are important in order to build the whole picture which were the findings of (Sims Gould & Martin Matthews, 2010).

In conclusion, the municipal FD recipient’s relatives should be seen as a powerful resource that can contribute to the development of the FD service Further the municipalities need to create the role of a person with overall responsibility for the FD service (for example a dietician) who should be the link between all of the involved professionals in the FD service, the consumers and their relatives (Table 2).

**Table 2:** Key findings

• Relatives are a powerful resource that can contribute to the development of the FD service • The legal limitations make it impossible for relatives to influence on the quality of the municipal FD service • Food preparation service in the home was promoted as more holistic and individual adjusted alternative than the distribution of ready-meals
